# Evidence of a predominance of sexual transmission of HTLV-1 in Salvador, the city with the highest prevalence in Brazil

**DOI:** 10.1186/1742-4690-12-S1-O3

**Published:** 2015-08-28

**Authors:** David Nunes, Ney Boa-Sorte, Maria Fernanda Rios Grassi, Kleber Pimentel, Maria Gloria Teixeira, Mauricio L Barreto, Inês Dourado, Bernardo Galvão-Castro

**Affiliations:** 1 Centro Integrativo e Interdisciplinar de HTLV, Escola Bahiana de Medicina e Saúde Pública;Salvador-Bahia-Brazil; 2 Laboratório Avançado de Saúde Pública, Centro de Pesquisas Gonçalo Moniz, Fundação Oswaldo Cruz, Salvador, Bahia, Brazil; 3 Instituto de Saúde Coletiva, Universidade Federal da Bahia, Salvador, Brazil

## 

Several investigations in Latin America have monitored health problems in urban spaces using what is called “sentinel areas” On the basis of this strategy, we detected an overall HTLV-1 prevalence of 1.7% in a representative sample of general population in Salvador, Northeast Brazil. In order to evaluate the sexual transmission of HTLV-1 in the general population of the city of Salvador, Northeast Brazil, the study analyzed a collection of 3,451 serum samples obtained by a simple random sampling procedure without replacement. Samples were in 30 “sentinel areas” f the city of Salvador, Bahia, Northeast Brazil collected from May 1998 to July 2000. HTLV-1 Syphilis and HIV infection were tested as a sexual transmission marker. Overall prevalence of HTLV-1 was 1.48% (51/3,451; 95% CI: 1.10% – 1.94%). Sixty-two percent of the seropositive individuals were women and the majority (65.3%) earned two minimum wages or less. Overall prevalence of syphilis and HIV was 26.67% (45/3,451; 95% CI 1.08 – 2.25) and 0.6% (21/3,451), respectively. Syphilis was present in 12 out of 51 (23.53%) individuals infected by HTLV-1 (26% of males and 19.35% of females), while only one person was infected with HIV (4.76%). HTLV-1 infection was associated with family income (OR 2.25; 95% CI 1.12 – 4.08), age (OR 9.58; 95% CI 5.01 – 18.29) and syphilis (OR 38.63; 95% CI 15.08 – 98.94). In the logistic regression analysis stratified by sex, HTLV-1 infection remained associated with age and syphilis diagnosis in males and with age, income and syphilis diagnosis in females. These data strongly suggest a predominance of heterosexual transmission of HTLV-1 in Salvador, Bahia. Furthermore, the majority of infected individuals were poor, had low levels of education and lived in worse living conditions, confirming our previous results.

